# Effects of paraoxonase activity and gene polymorphism on coronary vasomotion

**DOI:** 10.1186/2191-219X-1-27

**Published:** 2011-11-18

**Authors:** Vincent Dunet, Juan Ruiz, Gilles Allenbach, Paola Izzo, Richard W James, John O Prior

**Affiliations:** 1 Department of Nuclear Medicine, Centre Hospitalier Universitaire Vaudois (CHUV) and University of Lausanne, Rue du Bugnon 46, Lausanne, 1011, Switzerland; 2 Department of Endocrinology, Diabetology and Metabolism, Centre Hospitalier Universitaire Vaudois (CHUV) and University of Lausanne, Bugnon 46, Lausanne, 1011, Switzerland; 3 Clinical Diabetes Unit, Division of Endocrinology and Diabetology, University Hospital, 24, Rue Micheli-du-Crest, Geneva, 14, 1211 Switzerland

**Keywords:** paraoxonase, myocardial flow reserve, diabetes, rubidium-82

## Abstract

**Background:**

Paraoxonase 1 [PON1] is recognized as a protective enzyme against LDL oxidation, and PON1 polymorphism has been described as a factor influencing coronary heart disease [CHD] free survival. As coronary vasoreactivity is a surrogate of future cardiovascular events, we aimed at assessing the respective effect of the PON1 genotype and activity on coronary vasoreactivity in a population of type 2 diabetic patients.

**Methods:**

Nineteen patients with type 2 diabetes mellitus underwent ^82^Rb cardiac PET/CT to quantify myocardial blood flow [MBF] at rest, during cold pressor testing [CPT], and during adenosine-induced hyperaemia to compute myocardial flow reserve [MFR]. They were allocated according to Q192R and L55M polymorphisms into three groups (wild-type and LM/QR heterozygotes, MM homozygotes, and RR homozygotes) and underwent a measurement of plasmatic PON1 activity. Relations between rest-MBF, stress-MBF, MFR, and MBF response to CPT and PON1 genotypes and PON1 activity were assessed using Spearman's correlation and multivariate linear regression analysis.

**Results:**

Although PON1 activity was significantly associated with PON1 polymorphism (*p *< 0.0001), there was no significant relation between the PON1 genotypes and the rest-MBF, stress-MBF, or MBF response to CPT (*p *≥ *0.33*). The PON1 activity significantly correlated with the HDL plasma level (*ρ *= 0.63, *p *= 0.005), age (*ρ *= -0.52, *p *= 0.027), and MFR (*ρ *= 0.48, *p *= 0.044). Moreover, on multivariate analysis, PON1 activity was independently associated with MFR (*p *= 0.037).

**Conclusion:**

Our study supports an independent association between PON1 activity and MFR. Whether PON1 contributes to promote coronary vasoreactivity through its antioxidant activity remains to be elucidated. This putative mechanism could be the basis of the increased risk of CHD in patients with low PON1 activity.

## Background

Coronary heart disease [CHD] is the first cause of mortality in type 2 diabetic patients. Several risk factors have been recognized to contribute to the development of atherosclerotic lesions resulting in a decrease of coronary blood flow and myocardial ischemia. Among those factors, low high-density lipoprotein [HDL] plasma levels have emerged as one of the strongest predictor of CHD [1]. As a consequence, the mechanism by which HDL influences atherosclerosis has been extensively studied, and HDL has been shown to reduce oxidative stress and plaque formation. These antioxidant properties of HDL have been attributed to enzymes associated to HDL.

Paraoxonase 1 [PON1] is an enzyme exclusively located on HDL in serum [2]. PON1 hydrolyzes organophosphate substrates and metabolizes lipid peroxides leading to protect against accumulation of low-density lipoprotein [LDL] that contributes to atherosclerotic plaque formation. PON1 activity is in part determined by genetic polymorphism. Glutamine-192-arginine [Q192R] is a strong determinant of PON1 activity against exogenous substrates and has been associated with an independent cardiovascular risk [3,4]. Recent studies suggest that PON1 activity is more important than genotype to predict CHD [5,6]. However, the exact influence of PON1 genotype and activity on coronary blood flow remains uncertain. Malin et al. [7] showed that the PON1 genotype was neither significantly correlated with coronary blood flow response to adenosine stress nor with coronary flow reserve, both being recognized as surrogate markers of CHD. Interestingly, Yildiz et al. [8] found that indirect assessment of coronary blood flow on coronary angiography was associated with PON1 activity in a patient with a 'slow coronary flow' entity. Nevertheless, there is no evidence of a direct relation between PON1 activity and absolute coronary blood flow in type 2 diabetic patients.

Thus, we aimed at assessing the relation of PON1 genotype and activity to myocardial blood flow and myocardial flow reserve in a population of type 2 diabetic patients using ^82^Rb cardiac positron emission tomography/computed tomography [PET/CT].

## Methods

### Study design

In this monocentric study, patients with type 2 diabetes mellitus and PON1 polymorphism followed in the Department of Endocrinology, Diabetology and Metabolism of the University Hospital of Lausanne were prospectively enrolled from January to June 2009. Before inclusion, they all underwent a medical examination to screen for other cardiovascular risk factors: past or present smoking, hypertension (≥140/90 mmHg), LDL, HDL, and triglyceride [TG] levels, and family history of early CHD. Moreover, all patients with peripheral artery disease, known coronary artery disease or myocardial infarction, cardiomyopathy, renal failure, peripheral neuropathy, systemic disease or contraindication to adenosine (asthma, chronic obstructive bronchitis, second and third atrioventricular blocks) were excluded.

For every patient included, fasting glucose plasma, insulin plasma, LDL, HDL, TG, and high sensitivity C-reactive protein [hsCRP] levels were measured, and insulin resistance was assessed by calculating the homeostasis model assessment [HOMA-IR] index (HOMA-IR *= *fasting plasma glucose (mmol/L) × fasting plasma insulin (μU/mL)/22.5). The hsCRP/paraoxonase ratio was also computed. Patients refrained from any food for at least 6 h and from caffeine intake for ≥24 h before the PET studies. Every patient signed a written informed consent, and the study was approved by the ethics committee of the University of Lausanne.

### Paraoxonase 1 genotype and activity determination

PON1 polymorphisms in positions 192 (glutamine → arginine) and 55 (leucine → methionine) were genotyped by different methods. PON1 Q192R polymorphism was detected by polymerase chain reaction [PCR] amplification of specific alleles, and PON1 L55M polymorphism, by the restriction fragment length polymorphism method using the Hsp92II enzyme. Lymphocytes were isolated from the blood, and DNA was extracted using standard procedures. For PON1 Q192R genotyping, PCRs were performed on Robocycler^® ^Gradient 96 (Stratagene^®^, La Jolla, CA, USA) using primers described by Pinizzotto et al. [9]. It involved an initial denaturation at 95°C carried out for 5 min, followed by 35 cycles including denaturation at 95°C for 45 s, annealing at 58°C for 45 s, and elongation at 72°C for 1 min. The procedure was completed by a final incubation at 72°C for 7 min. For PON1 L55M genotyping, PCRs were carried out under the same conditions but for 28 cycles only. Fragments obtained were 500 bp long for the PON1-192 polymorphism, 384 bp long for the PON1-55 wild type, and 282 and 102 bp long for the PON1-55 mutant. All fragments were finally separated on a 2% agarose gel electrophoresis and visualized by ethidium bromide.

Serum PON1 activity was measured with paraoxon as substrate. Practically, the PON1 activity was measured by adding 20 μL of serum to a Tris buffer (100 mmol/L, pH 8.0) containing 2 mmol/L CaCl_2_and 5.5 mmol/L paraoxon (*O*,*O*-diethyl-*O*-*p*-nitrophenylphosphate; Sigma-Aldrich Co., St. Louis, MO, USA). The rate of generation of *p*-nitrophenol was determined over 3 min at 405 nm and 25°C, as previously described by James et al. [10].

### ^82^Rb cardiac PET/CT assessment

All patients underwent a series of three ^82^Rb cardiac PET/CT (Discovery LS, GE Healthcare, Milwaukee, WI, USA) studies. After a rest study, a cold pressor test [CPT] was carried out to assess myocardial blood flow [MBF] variations mainly due to endothelium-dependent vasomotion. CPT was done by a 2-min immersion of the left lower limb on ice water starting 1 min before the administration of ^82^Rb. Ten minutes afterwards, a pharmacological hyperemic stress was performed by adenosine infusion (140 μg/kg/min) over 6 min to measure a myocardial blood flow increase (stress-MBF) mainly due to endothelium-independent vasomotion and myocardial flow reserve (MFR = stress-MBF/rest-MBF), which also helped to exclude any underlying coronary artery disease. For each study, after a 10-s infusion of ^82^Rb (1450 MBq), a 6-min dynamic cardiac PET was acquired. Cardiac CT scans were also performed to correct for photon attenuation by soft tissues (before the rest study and just after the stress study). The good alignments between the PET and CT series were checked to avoid attenuation correction mistakes.

Data were processed with the full-automatic FlowQuant 1.2.3 software using a previously described one-tissue compartment modeling approach [11] to estimate the MBF at rest, during the cold pressure test, and during the pharmacological stress. Blood pressure, heart rate, and a 12-lead ECG were recorded at 1-min intervals during each procedure. To correct for cardiac workload, rest and CPT myocardial blood flows were normalized using the rate-pressure product (RPP = heart rate × systolic blood pressure).

### Statistical analysis

All statistical analyses carried out with Stata 10.1 continuous variables are presented as mean ± SD or as median (interquartile range, IQR). Allele frequencies were estimated by the gene-counting method, and Hardy-Weinberg's equilibrium was tested by chi-square test. To obtain a more meaningful genotype group size, patients were pooled into three groups: (1) wild-type, LM, and QR heterozygotes (group 1, *n *= 7); (2) MM homozygotes (group 2, *n *= 5); and (3) RR homozygotes (group 3, *n *= 6). Variable differences between these three genotype subgroups were assessed using one-way analysis of variance. Relations between variables were assessed using non-parametric Spearman's rank correlation (*ρ*). We secondly performed multivariate regression analysis (*β*) and stepwise multiple linear regression analysis to determine independent relationships to the PON1 activity or MBF, including all variables with significant correlations on univariate analysis. A *p *value < 0.05 was considered as statistically significant.

## Results

### Study population

In total, 19 patients (11 men, 8 women) with type 2 diabetes mellitus were enrolled. The clinical characteristics are summarized in Table 1. Among these patients, ten (53%) were wild-type, two (10%) were heterozygous, and seven (37%) were homozygous for Q192R polymorphism. Moreover, 11 (58%) were wild-type, 3 (16%) were heterozygous, and 5 (26%) were homozygous for L54M polymorphism. Both genotype distributions did not follow Hardy-Weinberg's equilibrium (*χ*^2^= 11.6 and 8.0, respectively; *p *< 0.01). All patients underwent the three PET/CT studies, and none had unexpected side effects during adenosine infusion. None had decreased stress-MBF < 2 mL/min/g or MFR < 2, thus excluding any hemodynamically significant coronary artery disease; no locally decreased myocardial perfusion imaging at rest was seen, excluding myocardial infarct. For one patient, PON1 activity measurement could not be subsequently measured on the blood sample. Laboratory, MBF, and MFR results of this patient were thus not included in subgroup comparisons.

**Table 1:** Study population characteristics

Variable (*n *= 19)	Mean ± SD or median (IQR) or *n *(%)
Age (years)	57.6 ± 9.8
Sex (% of women)	8 Women/11 men (42% women)
Weight (kg)	77 (66-94)
Body mass index (kg/m^2^)	25.9 (22.1-30.9)
Current smoking	3 (16%)
Hypertension	15 (80%)
Dyslipidemia	12 (63%)
Family history of early CHD	0 (0%)
Overall cholesterol (mmol/L)	4.2 ± 0.8
LDL-cholesterol (mmol/L)	2.3 ± 0.7
HDL-cholesterol (mmol/L)	1.2 ± 0.3
Triglyceride levels (mmol/L)	1.2 (0.9-1.8)
Fastening insulin (μU/mL)	10.8 (6.9-21.6)
Fastening glucose (mmol/L)	5.8 (5.4-7.5)
HOMA-IR (1)	3.3 (1.8-5.0)
hsCRP (mg/L, normal < 5 mg/L)	1.1 (0.4-2.8)
PON1 (U/L; *n *= 18)	79.3 ± 71.7
Arylesterase (U/L; *n *= 18)	41.0 (38.9-48.3)
Ratio hsCRP/PON1 × 1,000 (mg/U; *n *= 18)	47.1 (7.3-312)

IQR, interquartile range; CHD, coronary heart disease; LDL, low-density lipoprotein; HDL, high-density lipoprotein; HOMA-IR, homeostasis model assessment-insulin resistance; hsCRP, high sensitivity C-reactive protein.

### Relation to PON1 genotype

**Figure 1:**
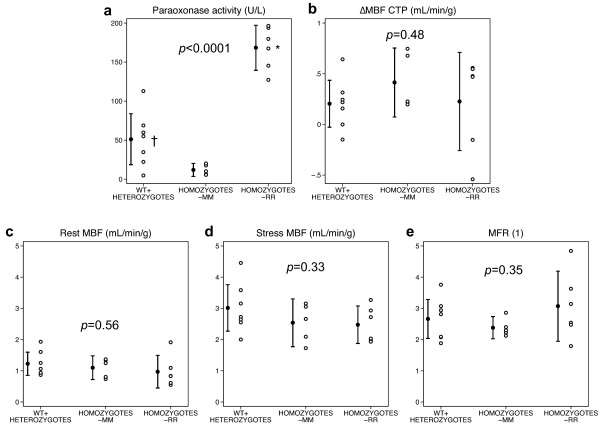
**Effect of paraoxonase genotype on paraoxonase activity and myocardial blood flow parameters**. Effect of paraoxonase genotype on (**a**) paraoxonase activity, (**b**) response to cold pressor testing (ΔMBF, increase in myocardial blood flow), (**c**) rest MBF, (**d**) stress MBF, and (**e**) MFR. Note that the paraoxonase genotype only had an effect on paraoxonase plasma levels (*p* < 0.0001), while there was no association with PET-measured indices of endothelium-dependent (ΔMBF) or -independent (stress MBF, MFR) vasomotion. Asterisks represent *p* < 0.0001 vs. wild type [WT] + heterozygotes and *p* < 0.0001 vs. homozygote-MM; dagger represents *p *= 0.083 vs. homozygotes-MM.

PON1 activity and laboratory results according to genotype subgroups are displayed in Table 2. Group 3 had a higher PON1 activity (168 ± 28 U/L) when compared with groups 1 (51 ± 35, *p* < 0.0001) and 2 (11.9 ± 6.7, *p* < 0.0001; Figure 1a), and there was a trend for a difference between groups 1 and 2 (*p *= 0.083). Arylesterase activity was not statistically different according to the PON1 genotype (*p *= 0.22). None of the common biological variables were significantly influenced by the PON1 genotype. Moreover, we did not find any significant difference for rest-MBF, CPT-MBF, MBF difference between CPT and rest, stress-MBF or MFR between groups 1, 2, and 3 (Table 3, Figure 1b,c,d,e).

**Table 2:** Laboratory analyses according to paraoxonase genotype subgroups

Variable (*n *= 18)	Group 1^a^ (*n *= 7)	Group 2^b^ (*n *= 5)	Group 3^c^ (*n *= 6)	*p *value*
LDL-cholesterol (mmol/L)	2.3 ± 0.9	2.5 ± 0.6	2.1 ± 0.3	0.71
HDL-cholesterol (mmol/L)	1.1 ± 0.3	1.0 ± 0.1	1.3 ± 0.3	0.16
Triglyceride levels (mmol/L)	2.1 ± 0.7	1.7 ± 0.8	2.1 ± 2.5	0.90
Fastening insulin (μU/mL)	26.2 ± 15.0	23.2 ± 17.9	40.3 ± 55.0	0.67
Fastening glucose (mmol/L)	8.3 ± 3.7	8.7 ± 2.5	7.7 ± 2.9	0.86
HOMA-IR (1)	11.0 ± 10.9	8.8 ± 6.8	18.6 ± 32.8	0.70
hsCRP (mg/L, normal < 5 mg/L)	5.6 ± 7.1	5.6 ± 5.7	1.6 ± 1.9	0.36
PON1 activity (U/L)	51.1 ± 35.3	11.9 ± 6.7	168 ± 27	<0.0001
Arylesterase (U/L)	46.5 ± 14.0	36.2 ± 8.9	45.9 ± 5.5	0.22
Ratio hsCRP/PON1 × 1,000 (mg/U)	365 ± 774	401 ± 254	10.0 ± 12.5	0.37

*p Values were calculated using one-way analysis of variance. ^a^Group1 = wild type + LM/QR heterozygotes; ^b^group 2 = MM homozygotes; ^c^group 3 = RR homozygotes; PON1, paraoxonase 1; LDL, low-density lipoprotein; HDL, high-density lipoprotein; HOMA-IR, homeostasis model assessment- insulin resistance; hsCRP, high sensitivity C-reactive protein; MM, methionine-methionine; RR, arginine-arginine.

**Table 3:** Myocardial blood flow values according to paraoxonase genotype subgroups

Variable (*n *= 18)	Group 1^a^ (*n *= 7)	Group 2^b^ (*n *= 5)	Group 3^c^ (*n *= 6)	*p *value*
Rest-MBF (mL/min/g)	1.2 ± 0.4	1.1 ± 0.3	1.0 ± 0.5	0.56
CPT-MBF (mL/min/g)	1.4 ± 0.5	1.5 ± 0.5	1.2 ± 0.3	0.48
MBF difference CPT-rest (mL/min/g)	0.2 ± 0.3	0.4 ± 0.3	0.2 ± 0.5	0.55
MBF difference CPT-rest (%)	18 ± 17	36 ± 16	40 ± 51	0.46
Stress-MBF (mL/min/g)	3.0 ± 0.8	2.5 ± 0.6	2.5 ± 0.6	0.33
MFR (1)	2.7 ± 0.7	2.4 ± 0.3	3.1 ± 1.1	0.35

*p Values were calculated using one-way analysis of variance. ^a^Group1 = wild type + LM/QR heterozygotes; ^b^group 2 = MM homozygotes; ^c^group 3 = RR homozygotes. CPT, cold pressor test; MBF, myocardial blood flow; MFR, myocardial flow reserve; MM, methionine-methionine; RR, arginine-arginine.

### Relation to PON1 activity

PON1 and arylesterase activities were both strongly associated with HDL plasma level (*ρ *= 0.63, *p *= 0.005 and *ρ *= 0.71, *p *= 0.001, respectively). PON1 activity was also correlated with age (*ρ *= -0.52, *p *= 0.027) and with arylesterase activity (*ρ *= 0.61, *p *= 0.008). Moreover, there was a trend for a negative correlation between hsCRP and PON1 activity (*ρ *= -0.36, *p *= 0.14). Including significant univariate predictors (age, HDL, arylesterase activity, and MFR), the multivariate linear regression analysis revealed that HDL (*p *= 0.04) was independently related to the PON1 activity (Table 4). Likewise, using the same univariate predictors, stepwise multiple linear regression analysis highlighted that both HDL (*p *= 0.015) and MFR (*p *= 0.037) were independently associated with the PON1 activity.

**Table 4:** Univariate (*ρ*) and multivariate (*β*) correlations between PON1 activity and study population characteristics

Variable (*n *= 18)	Univariate		Multivariate	
	*ρ*	*p *value	*β*	*p *value
Age	-0.52	0.03	-0.28	0.3
Sex	0.08	0.8		
Weight	-0.33	0.18		
Body mass index	-0.43	0.07		
Overall cholesterol	0.08	0.7		
LDL-cholesterol	0.01	1.0		
HDL-cholesterol	0.63	0.005	0.52	0.04
Triglyceride	-0.24	0.33		
Insulin	0.11	0.65		
Glucose	-0.17	0.5		
HOMA-IR	0.03	0.9		
hsCRP	-0.36	0.14		
Arylesterase	0.61	0.008	-0.05	0.8
Rest-MBF	0.03	0.9		
Stress-MBF	0.15	0.5		
MFR	0.48	0.04	0.34	0.2
CPT-MBF	-0.18	0.47		
MBF difference CPT-rest	-0.17	0.5		

Relations between variables were assessed using non-parametric Spearman's correlation coefficients (*ρ*). Independent relations were assessed using multivariate regression analysis (*β*). PON1, paraoxonase 1; LDL, low-density lipoprotein; HDL, high-density lipoprotein; HOMA-IR, homeostasis model assessment- insulin resistance; hsCRP, high sensitivity C-reactive protein; MBF, myocardial blood flow; MFR, myocardial flow reserve; CPT, cold pressor test.

**Figure 2:**
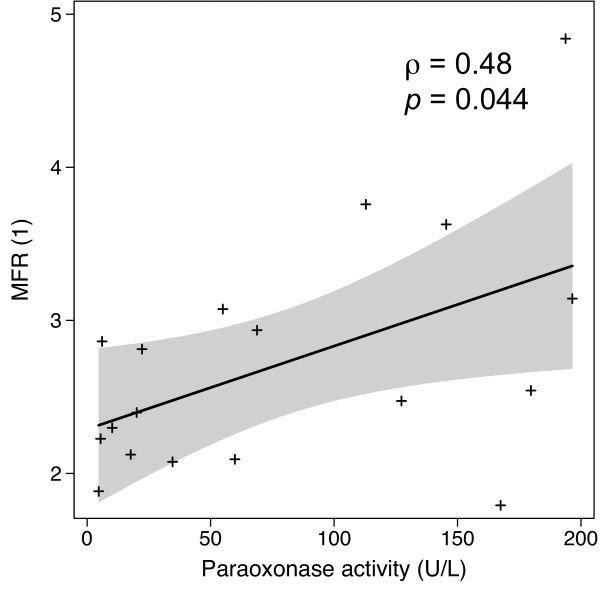
**Paraoxonase activity effect on MFR showing an association between increased paraoxonase level and better MFR**. The gray shading represents the 95% confidence area.

Regarding myocardial flow quantitation, we found no significant correlation between myocardial blood flow at rest, at stress, or myocardial blood flow response to CPT and patients' characteristics depicted in Table 1. However, on univariate analysis, myocardial flow reserve was correlated with PON1 activity only (*ρ *= 0.48, *p *= 0.044, Figure 2).

## Discussion

Since CHD is the first cause of mortality in type 2 diabetic patients, cardiovascular risk factors have been extensively studied to improve the understanding of atherosclerosis and mechanisms leading to the development of coronary artery disease. Whereas PON1 genotypes and activities have been described as independent predictors of CHD [5,6], there was no evidence of a reduction of hyperemic MBF. Thus, our study is the first report of an independent relation between PON1 activity and MFR assessed by cardiac PET/CT.

Owing to the need of a better understanding of atherosclerosis development and protective factors, the role of HDL has been extensively studied and is known as one of the strongest protectors against coronary artery disease [1]. Consequently, the influence of PON1 polymorphism as a main component of the HDL complex was assessed. Among several polymorphisms, Q192R and L55M emerged as the most interesting [3]. PON1 192R and PON1 55L were reported as more efficient in decreasing hydrolysis of lipid peroxides by promoting PON1 activity [4]. Our data confirm that PON1 activity is significantly different according to PON1 genotypes (*p* < 0.0001). The PON1 activity of the MM genotype was low (11.9 ± 6.7 U/L), but this may be due to the small number of subjects (*n *= 5) and to the fact that MM patients in our study are all QQ homozygotes, which is an additional genetic factor that lowers paraoxonase activity.

Studies aiming at assessing the predictive value of PON1 polymorphism found controversial results. Whereas a few studies reported that PON1 R allele was independently related to CHD, others failed to show it [12]. A recent study by Acampa et al. found no difference in genotype between CAD-suspected patients with and without ischemia undergoing cardiac SPECT [13]. This highlights the limits of the genotyping approach that conceals external influence upon enzyme function. For instance, in our study, age was correlated with PON1 activity (*ρ *= -0.52, *p *= 0.027) that sustains the hypothesis of an age-dependent decrease of PON1 activity [14], which may be due to the development of oxidative stress conditions with aging such as systemic inflammation, leading to an increased risk of CHD.

MBF and MFR both have predictive values of cardiovascular event-free survival [15,16]. According to genotype, we found no difference of MBF at rest, during the CPT, or at stress. Pasqualini et al. reported a correlation between PON1 activity and peripheral endothelium-dependent vasoreactivity in patients with peripheral artery disease [17]. Although they performed a flow-mediated dilation measurement with good intra-observer reproducibility, this technique presents a high variability [18] that may be a concern in reproducing such results. Using similar highly reproducible PET/CT methods [19] such as that used in our study, Malin et al. found no difference of hyperemic MBF between genotype groups in a population of 49 young healthy men [7]. Our study extends their results in a patient population with type 2 diabetes, but not with other associated health conditions where we did not find any difference in response to adenosine or CPT according to genotype. Nor was there any correlation between PON1 activity and CPT-MBF, suggesting that PON1 is not involved in atherosclerosis by an impairment of endothelium-dependent coronary vasoreactivity. Regarding PON1 activity rather than PON1 genotype, we found an independent correlation between PON1 activity and MFR (*p *= 0.037). In several studies, PON1 192R was described as an independent cardiovascular risk factor [12]. Mackness et al. [5] highlighted in a 417-patient population compared with 282 control subjects that not PON1 Q192R polymorphism, but PON1 activity was significantly lower in patients experiencing CHD. Moreover, Bhattacharyya et al. [20] brought to light that PON1 activity independently predicted major adverse cardiac event-free survival. Though we report a positive association between PON1 activity and MFR, the exact influence of PON1 on mainly endothelium-independent coronary vasoreactivity remains unclear. Whether PON1 may concur in modifying MFR needs to be investigated further. It could constitute a putative mechanistic link to clarify the predictive value of PON1 activity on CHD occurrence. This association may be of importance in type 2 diabetic patients who have decreased levels of HDL cholesterol.

Although we report for the first time a direct relation between MFR and PON1 activity, our study presents some limitations. We decided to focus on patients with type 2 diabetes mellitus whose genotype was already known. Our study was carried out in a selected, small population of patients with type 2 diabetes mellitus, hence resulting in deviations from Hardy-Weinberg's equilibrium. Regardless, our results need to be confirmed in a larger prospective cohort of patients with type 2 diabetes mellitus. The absence of correlation between the MBF response to adenosine or CPT regarding the PON1 genotype or PON1 activity confirms the results of Malin et al.[7] and would be in agreement with the study of Acampa et al. [13]. This seems to indicate that PON1 is not involved in the development of atherosclerosis by an impairment of endothelium-dependent vasomotion, but the exact mechanism remains unknown. Furthermore, PON1 activity variations may be a part of a multifactorial mechanism leading to a decreased coronary vasoreactivity. The relative effect of PON1 on coronary vasomotion as well as its relative value in predicting cardiac event-free survival remains to be determined.

Lastly, a power analysis indicates that the proposed patient allocation into three groups of paraoxonase genotype would have allowed the showing of ≥50% differences in MBF or MFR according to genotype (type I error *α *= 0.1, power 1-*β *= 0.8), which were not observed. However, smaller differences might have been missed by the present study due to the small population size. Thus, smaller genotype-related effects cannot be excluded by our study, and larger multicenter studies would be needed to exclude such an effect.

As cardiac PET/CT has the ability to detect early MFR modification under therapy, this may help in investigating new PON1 activity-enhancing combinations of nicotinic acid and laropiprant, such as those currently used in the HPS2-THRIVE [21].

## Conclusion

Our study demonstrates an association between PON1 activity and MFR in type 2 diabetic patients though the exact mechanism by which PON1 influences MFR remains unclear. Our study also shows no evidence of PON1 influencing endothelium-dependent vasoreactivity. The mechanism linking PON1 activity and MFR remains to be determined though. This might open new perspectives for treatments aiming to improve MFR by promoting PON1 activity.

## Abbreviations

BMI: body mass index; CHD: coronary heart disease; CPT: cold pressor test; ECG: electrocardiogram; HDL: high-density lipoprotein; HOMA-IR: homeostasis model assessment-insulin resistance index; hsCRP: high sensitivity C-reactive protein; IQR: interquartile range; LDL: low-density lipoprotein; MBF: myocardial blood flow; MFR: myocardial flow reserve; PET/CT: positron emission tomography/computed tomography; PON1: paraoxonase 1; RPP: rate pressure product; SD: standard deviation; TG: triglyceride.

## Competing interests

VD, JR, GA, PI and RWJ declare that they have no competing interests. JOP has received a scientific grant support for this project from Bracco Diagnostics Inc., P.O. Box 5225, Princeton, NJ 08543-5225, the manufacturer of the Cardiogen-82^^®^^, the ^82^Rb generator used in this study for performing the PET/CT examinations.

## Authors' contributions

VD has been involved in data acquisition, analysis and interpretation, in drafting and revising the manuscript. JR has been involved in the study design, data acquisition and interpretation, and in revising the manuscript. GA has been involved in the study design and in revising the manuscript. PI has been involved in data acquisition and in revising the manuscript. RWJ has been involved in the study design, data acquisition, and in revising the manuscript. JOP has been involved in the study design, data acquisition, analysis and interpretation, and in revising the manuscript. All the authors gave their final approval for publication.
